# New Nanobiocomposite Materials for Bioelectronic Devices

**Published:** 2015

**Authors:** D. V. Pankratov, E. González-Arribas, Yu. M. Parunova, M. A. Gorbacheva, Yu. S. Zeyfman, S. V. Kuznetsov, A. V. Lipkin, S. V. Shleev

**Affiliations:** National Research Center “Kurchatov Institute”, Akademika Kurchatova Sq., 1, Moscow, 123182, Russia; A.N. Bach Institute of Biochemistry, Russian Academy of Sciences, Leninsky prospect, 33, building 2, Moscow, 119071, Russia; Malmö Univeristy, Jan Waldenströms gata, 25, Malmö, 21428, Sweden; I.G. Petrovsky Bryansk State University, Bezhitskaya Str., 14, Bryansk, 241036, Russia

**Keywords:** glucose oxidase, graphene, conducting organic polymer, carbon nanotubes, nanobiocomposite/double function electrode

## Abstract

We have developed and synthesized nanobiocomposite materials based on graphene, poly(3,4-ethylenedioxythiophene), and glucose oxidase immobilized on the surface of various nanomaterials (gold nanoparticles and multi-walled carbon nanotubes) of different sizes (carbon nanotubes of different diameters). Comparative studies of the possible influence of the nanomaterial’s nature on the bioelectrocatalytic characteristics of glucose- oxidizing bioanodes in a neutral phosphate buffer solution demonstrated that the bioelectrocatalytic current densities of nanocomposite-based bioanodes are only weakly dependent on the size of the nanomaterial and are primarily defined by its nature. The developed nanobiocomposites are promising materials for new bioelectronic devices due to the ease in adjusting their capacitive and bioelectrocatalytic characteristics, which allows one to use them for the production of dual-function electrodes: i.e., electrodes which are capable of generating and storing electric power simultaneously.

## INTRODUCTION

Nanobiocomposite materials are increasingly in use in various fields of science and technology, including new biomedical technologies [1]. Modern bioelectronic nanocomposite-based devices (biosensors, biofuel elements, biobatteries, etc.) can be used for continuous monitoring of an organism’s state, for targeting organs and tissues, as well as for spot delivery of drugs. Comparative studies of the particular features of the performance of nanobiocomposites in buffer solutions and complex human physiological fluids provide the foundation for the development of highly efficient and stable bioelectronics for biomedical applications. This work discusses the production of novel nanobiocomposite materials based on graphene, poly(3,4-ethylenedioxythiophene), and glucose oxidase immobilized on the surface of nanomaterials of different nature (gold nanoparticles and multi-walled carbon nanotubes) and sizes (carbon nanotubes of different diameters) and examines their properties under near-physiological conditions.

## MATERIALS AND METHODS

**Materials and Methods**

Na_2_HPO_4_·2H_2_O, NaH_2_PO_4_·H_2_O, NaCl, HAuCl_4_·3H_2_O, H_2_SO_4_, LiClO_4_, sodium citrate, acetonitrile (≥ 99.9%, ACN), toluene (≥ 99.8%), gelatin (GE), *D*-glucose, 3,4-ethylenedioxythiophene (EDOT), polyethylene glycol (PEG), 25% glutaraldehyde solution (GA), tetrathiafulvalene (TTF), 7,7,8,8-tetracyanoquinodimethane (TCNQ), tetrahydrofuran (THF), and glucose oxidase (GOx) from *Aspergillus niger *were purchased from Sigma-Aldrich (USA) and used without further purification. Ethanol (95%) and argon were purchased from Kemetyl AB (Sweden) and AGA Gas AB (Sweden), respectively. All solutions were prepared using deionized water (18 MΩ∙cm) produced using a PURELAB UHQ II system from ELGA Labwater (UK).

Nanobiocomposites were synthesized using gold nanoparticles (AuNP) with a diameter of 20 nm and three types of carbon nanomaterials: graphene (GR, 1.6 nm thick, less than three carbon monolayers) and two types of multi-walled carbon nanotubes (CNT): CNT_1_ (outer diameter of 10–15 nm, inner diameter of 2–6 nm, length of 0.1–10 μm) and CNT_2_ (outer diameter of 20–30 nm, inner diameter of 1–2 nm, length of 0.5–2 μm). GR was purchased from Graphene Supermarket (USA); CNT, from Sigma-Aldrich (USA). AuNP were synthesized by the method described in [2], using sodium citrate as a reductant. 50 mL of a 250 μM HAuCl_4_ solution was brought to a boil under constant stirring, and 750 μL of an aqueous 1 wt. % sodium citrate solution was added. After the addition of sodium citrate, the mixture was incubated for 10 min under constant stirring without heating. The resultant AuNP suspension was cooled to room temperature and concentrated (50-fold) by centrifugation at 10,000 *g *for 30 min [3]; 98% of the supernatant was removed, and the AuNP precipitate was re-suspended using sonication.

Electrochemical measurements were performed using a μAutolab Type III/FRA2 potentiostat/galvanostat (Metrohm Autolab BV, Netherlands) using a three-electrode circuit with a saturated calomel reference electrode (242 mV vs. normal hydrogen electrode, NHE) and a platinum wire as an auxiliary electrode. All potentials are reported with respect to SCE, unless specified otherwise.

Sonication was performed on an Ultrasonic Cleaner XB2 bath (VWR International Ltd., UK). Scanning electron microscopy (SEM) images were obtained using a EVO LS 10 high-resolution scanning electron microscope from Zeiss (Germany).

**Production of nanobiocomposite material-based electrodes**

Polycrystalline gold disc electrodes from Bioanalytical Systems (USA) with a geometric surface area of 0.031 cm^2^ were mechanically cleaned through polishing with Microcloth paper (Buehler, UK) in an aluminum oxide suspension with a particle size of 0.1 μm (Struers, Denmark). The electrodes were further washed with deionized water, sonicated in ethanol for 5 min to remove residual aluminum oxide particles, and electrochemically cleaned through cycling in 0.5 M H_2_SO_4_ using a range of potentials from –0.2 to +1.7 V for 20 cycles at a scan rate of 0.1 V/s, washed with water, and dried in an airflow.

**Fig. 1:**
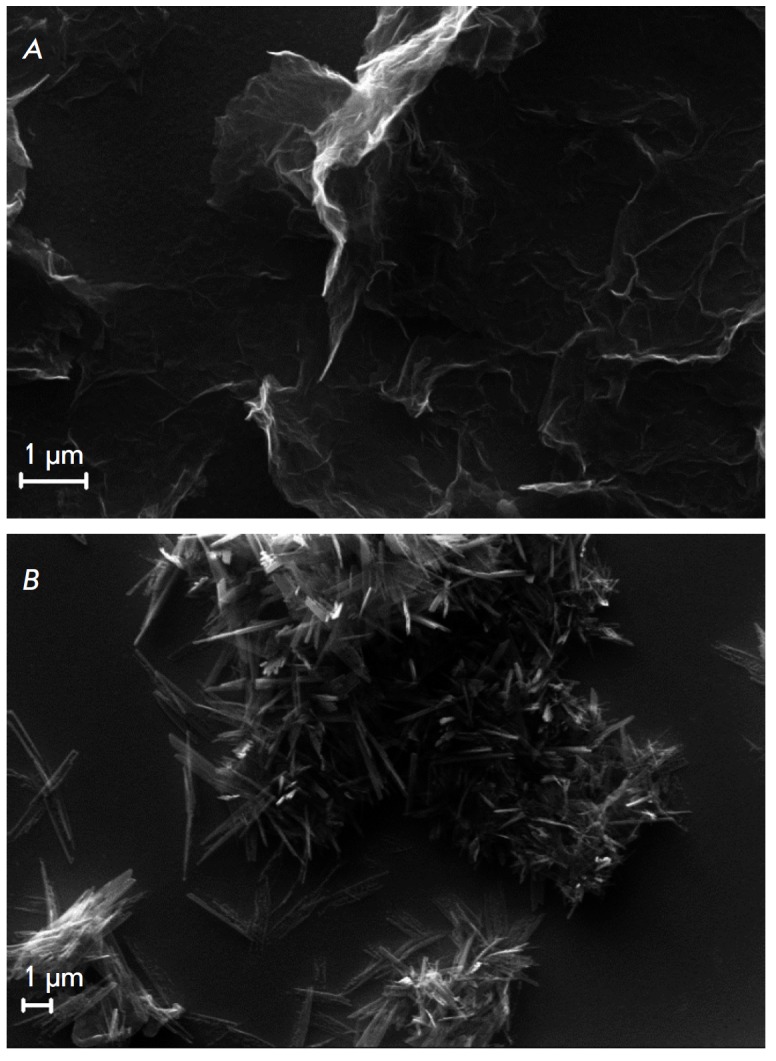
SEM images of the surfaces of (A) Au|PEDOT/GR and (B) Au|PEDOT/GR|TCNQ/TTF electrodes

Poly(3,4-ethylenedioxythiophene)/graphite (PEDOT/GR) nanocomposite was synthesized on Au surface by potentiodynamic cycling in a range of potentials from 200 to 1,300 mV (1 cycle at 100 mV/s) in 0.1 M phosphate buffered saline (PBS, pH 7.4) containing 20 mM EDOT, 1 mM PEG, 0.1 M LiClO_4_ and GR at a GR : EDOT weight ratio of 1:5 [4]. Prior to electropolymerization, the mixture was sonicated for 1 h to obtain a stable suspension and then purged with argon for 20 min to remove oxygen. The resulting layer of PEDOT/GR was sufficiently homogeneous with only minor defects filled with polymer (*Fig. 1A*).

The charge-transfer complex (CTC) TCNQ/TTF, a known mediator to ensure contact between the electrode and the immobilized GOx (see below), was synthesized on the surface of the composite PEDOT/GR material [4]. TCNQ and TTF were dissolved in THF and ACN, respectively, to obtain solutions with a concentration of 1.2 mg/mL. The TCNQ (1 μL) and TTF (2 μL) solutions were mixed on the surface of the PEDOT/GR composite; the excess unreacted TTF was washed away with ACN after the CTC crystallization process had been completed. The resulting CTC crystals had a characteristic needle-like shape, which is in accordance with the data in [5], but were rather unevenly distributed over the electrode surface, forming islands corresponding to the crystallization centers (*Fig. 1B*).

2 μL of nanomaterial suspension (CNT or AuNP) was applied to the surface of the TCNQ/TTF complex. To obtain a stable suspension, 1 mg of CNT was mixed with 1 mL of toluene; the AuNP concentrate was 10- fold diluted with deionized water; the mixtures obtained were sonicated for 20 min.

**Fig. 2:**
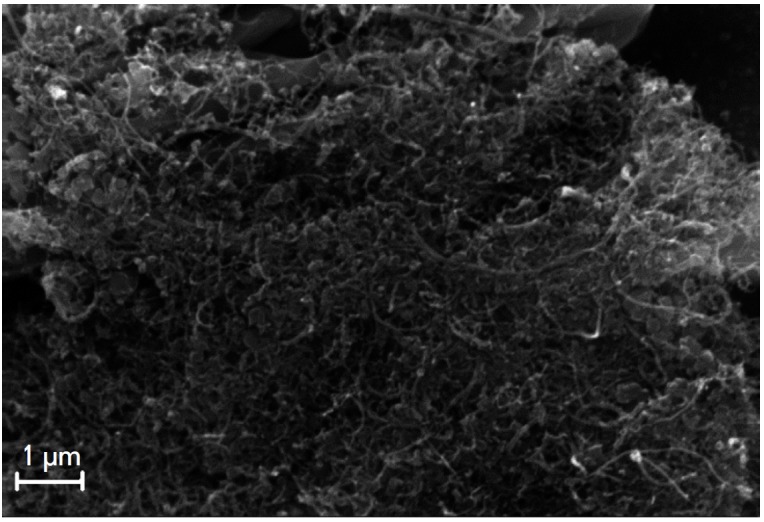
SEM image of the surface of the Au|PEDOT/ GR|TCNQ/TTF|CNT_1_/GOx electrode

To perform biomodification of the resulting nanobiocomposites, PEDOT/GR|TCNQ/TTF|CNT and PEDOT/GR|TCNQ/TTF|AuNP, 2 μL of a GOx solution (10 mg/mL in PBS) was applied to the electrode surface and kept at +4 °C for 1 h. To evaluate the influence of the nanomaterial on the biocatalytic properties of the electrode, a PEDOT/GR|TCNQ/TTF|GOx biocomposite was produced with the enzyme immobilized directly on the CTC surface. The SEM image of the PEDOT/ GR|TCNQ/TTF|CNT1/GOx nanobiocomposite surface is shown in Fig. 2. Remarkably, the surface is well developed and the CNT1/GOx layer is sufficiently homogeneous and evenly coats the CTC.

2 mL of a gelatin solution in water (2.5 wt. %) was applied to the electrode surface to stabilize the resultant structure, followed by drying for 1 h at room temperature. The electrodes were subsequently immersed in an aqueous GA solution (5 wt. %) for 60 s and washed with water.

## RESULTS AND DISCUSSION

**Fig. 3:**
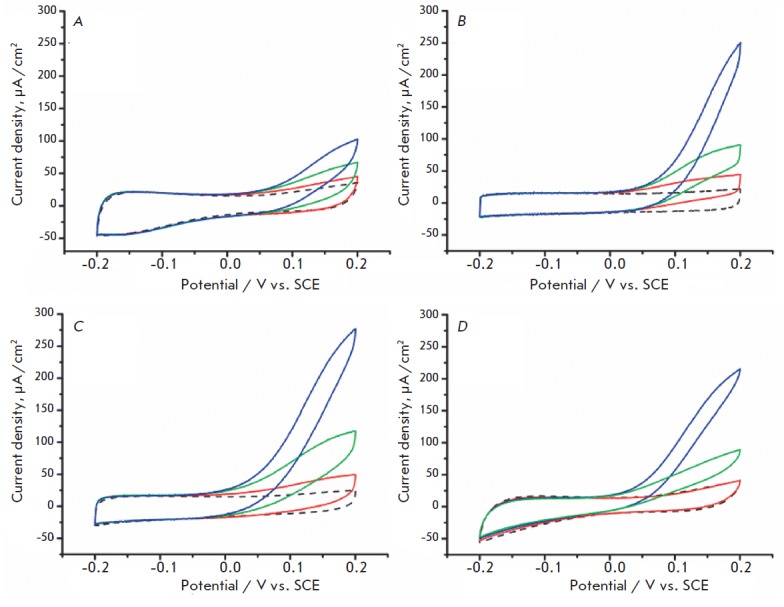
CVs of bioanodes submerged in PBS. Au|PEDOT/GR|TCNQ/TTF|GOx|GE (A), Au|PEDOT/GR|TCNQ/TTF|Nanomaterial/ GOx|GE (B-D), PB without glucose (dashed line), and PB with glucose (solid line), mmol L^-1^. 0.05 (red), 5 (green) and 50 (blue). Nanomaterial: CNT_1_ (B), CNT_2_ (B), AuNP (D)

The biocatalytic properties of the designed electrodes were studied in 0.1 M PB in a range of potentials from –0.2 to 0.2 V relative to the SCE at a scan rate of 10 mV/s. The cyclic voltammograms (CV) of the bioanodes with different structures are shown in Fig. 3.

The capacity of the produced electrodes is independent of the presence of nanocomposite and ranges from 1.5 to 2.5 mF/cm^2^ for all types of structures. It should be noted that it is easy to modify the capacity of the nanobiocomposites both during the PEDOT/GR synthesis (the number of electropolymerization cycles) and when designing nanobiocomposites. This feature allows one to use the developed materials to design and optimize hybrid bioelectrodes with dual functions: generation and storage of electrical power [6].

The data show that a pronounced bioelectrocatalytic response with an initial potential of glucose electrooxidation ca. 0 V, increasing with the glucose concentration rising to 50 mM, was recorded for all electrodes in glucose-containing PBS, which is consistent with the published data for CTC/GOx systems [7].

The biocatalytic current density ( *j*) of Au|PEDOT/GR|TCNQ/TTF|GOx|GE electrodes was low compared to the samples containing nanomaterial, which can be attributed to the uneven distribution of CTC over the electrode surface (*Fig. 1B*). Enzyme adsorption on the CTC surface blocks the mediating electron transfer for the GOx molecules adsorbed onto the PEDOT/GR nanocomposite and, therefore, reduces the proportion of the bioelectrochemically active enzyme.

In the case of Au|PEDOT/GR|TTF/TCNQ|nanomaterial/ GOx|GE electrodes, the experimental value of *j* was 229 ± 13 and 251 ± 15 μA/cm2 for CNT_1_ and CNT_2_ as a nanomaterial, respectively, and 175 ± 8 μA/cm2 for AuNP, under conditions similar to those for bioanodes containing no nanomaterials. The *j *value was ca. 10% higher for CNT_2_-based electrodes than for those based on CNT1, which is in agreement with the difference in the capacity of Au|PEDOT/GR|TCNQ/TTF|CNT1/ GOx|GE and Au|PEDOT/GR|TCNQ/TTF|CNT2/GOx/ GE (1.63 ± 0.05 and 1.85 ± 0.05 mF/cm^2^, respectively). The higher *j *values in the case of CNT_2_ can be attributed to a larger specific surface area rather than to better conditions for enzyme immobilization. This fact is consistent with the data obtained previously for bilirubin oxidase adsorbed onto the surface of modified AuNP with different diameters exceeding the enzyme size [8].

## CONCLUSIONS

Our research resulted in the development of multicomponent nanobiocomposites with the possibility of controlled regulation of their capacitive and bioelectrocatalytic parameters. The material obtained can be used to create modern bioelectronic devices which are fully operational under near-physiological conditions.

## Abbreviations

ACN: acetonitrile
GE: gelatin
EDOT: 3,4-ethylenedioxythiophene
PEDO: poly(3,4-ethylenedioxythiophene)
PEG: polyethylene glycol
GA: glutaraldehyde
TTF: tetrathiafulvalene
TCNQ: 7,7,8,8-tetracyanoquinodimethane
THF: tetrahydrofuran
GOx: glucose oxidase
AuNP: gold nanoparticles
GR: graphene
CNT: carbon nanotubes
SCE: saturated calomel electrode
SEM: scanning electron microscopy
Au: gold electrode
CTC: charge transfer complex
PB: phosphate buffer
j: bioelectrocatalytic current density
CV: cyclic voltammogram
